# Experimental and Numerical Studies on Fixed Steel Sheets Subjected to Underwater Explosion

**DOI:** 10.3390/ma15186419

**Published:** 2022-09-15

**Authors:** Si Chen, Jian Qin, Shuo Deng, Xiangyao Meng, Ruiyuan Huang, Xiaoqiang Yang

**Affiliations:** 1 Beijing Institute of Technology, School of Mechatronical Engineering, Beijing 100081, China; 2 Naval Research Academy, Beijing 100161, China; 3 College of Civil Engineering, Fuzhou University, Fuzhou 350116, China

**Keywords:** underwater explosion, steel sheet, shock wave, bubble pulsation, finite element analysis

## Abstract

This study presents underwater explosion tests with three different TNT charge weights to investigate the dynamic responses of a fixed steel sheet. A finite element model was established and benchmarked by comparing the bubble development and deformation distribution from the tests. The steel sheet shows a deformation process of hogging, sagging, and hogging again, due to the actions of shock waves, bubble expansion, bubble collapse, and bubble pulsation. The air may be sucked into the bubble during the hogging process, making the bubble collapse earlier and resulting in a relatively lower sagging deformation for large charge weights of TNT. The deformation caused by bubble pulsation is larger than that by the shock waves, owing to the large time duration of bubble pulsation. A parametric analysis was conducted to study the influence of steel grade, plate thickness, detonation distance, and the shape and position of charges on the dynamic behavior of steel plates subjected to underwater explosions. It shows that the damage to the steel plate gradually decreases, with the increase in steel strength, plate thickness, and detonation distance. The influence of the shape and position of charges is limited. The largest deformation is observed when the detonation distance increases to bubble radius.

## 1. Introduction

Underwater explosion presents a complicated process [1,2,3], differing from explosions in the air. The shock wave from an underwater explosion propagates over a very long distance and maintains its destructive ability, since the density of water is 1000 times that of air. In the case of mid-field and far-field explosions, only the shock wave pressure is considered [4,5,6]. In the case of a near-field explosion, the shock wave is first produced when the explosive is detonated underwater, and it will reflect when it propagates to contact the free liquid surface and the structural surface, causing the pressure of the nearby water to drop sharply. A cavitation effect will occur when the water pressure drops to the cavitation limit [7,8]. Subsequently, the high-temperature and high-pressure detonation products form a bubble in the water [9,10,11]. The movement of the bubble causes the flow of water, which is generally defined as a retarded flow [12,13,14]. When the bubble expands to its maximum volume, it begins to shrink. High-velocity water jets are formed during shrinkage [15,16]. After the bubble shrinks to a minimum volume, it will rebound to form a pulsating pressure [17]. The structural damage caused by underwater explosions is usually severe, and the deformation development and failure mechanism of structures subjected to bubble pulsation and water jets during near-field explosions are complicated [11,18,19,20]. Hence, studies on underwater explosions are always research hotspots.

As the most common components of ships or marine structures, steel plates may be locally damaged by underwater explosions, resulting in overall failure in some cases. Hence, a steel plate subjected to underwater explosions needs to be systematically investigated to clarify the deformation development and failure mechanism, and finally provide a reference for anti-explosion design. Many researchers have investigated the damage effects of plate structures subjected to underwater explosions. Rajendran et al. [6] investigated the damage to a circular plate subjected to underwater explosions, and a new prediction model has been proposed by considering the input shock energy, deformation contour, material properties, and plate thickness. Jiang et al. [21] conducted experimental tests to study the dynamic responses of pre-cracked aluminum plates subjected to underwater explosions. Leblanc et al. [22] numerically studied the dynamic responses of curved composite plates subjected to underwater explosions. Results showed that the deformation of the plate is significantly affected by the plate curvature, plate thickness, and thickness distribution. Jin et al. [23] numerically investigated the interaction between an underwater explosion bubble and a moveable plate with the basic characteristics of a sandwich structure using the boundary element method (BEM). Zhang et al. [24] calculated the dynamics of an underwater explosion bubble near elastic–plastic boundaries by combining the BEM and the finite element method (FEM). The results indicated that the damages caused by the retarded flow, pulsating pressure, and jetting load on the structures cannot be ignored.

However, the existing research still has limitations: (1) some research only investigated the behavior caused by the shock waves, without considering the subsequent bubble pulsation and water jets which typically also resulted in severe damage to structures; (2) for those studies on the dynamic responses caused by bubble pulsation, existing methods typically ignored the effect of shock waves, and separately considered the actions of shock waves and bubble pulsation [25,26,27]. Those investigations adopted the BEM, which assumed that a high-pressure bubble was generated after the explosion, and the initial radius and pressure of the bubble were set to simulate the bubble pulsation process, without simulating the shockwave propagation; (3) The response of the elastic–plastic boundary in the whole process of the underwater explosion had not been investigated deeply. Therefore, a systematic study on the dynamic response of steel plates under the coupling action of shock waves, retarded flow, bubble pulsation, and water jets (i.e., the whole process of underwater explosion) is urgent, to further clarify the deformation development and failure mechanism.

In this study, underwater explosion tests with three TNT equivalents were carried out to investigate the dynamic responses of a fixed square steel sheet. The whole process, including impulsive pressure of shock waves and bubble pulsation, and final deformation, was experimentally obtained. A finite element (FE) model was established using a coupled Eulerian–Lagrangian (CEL) method [28]. Based on the obtained experimental results, the established FE model was benchmarked by comparing the whole development process of the bubble and deformation distribution. The mechanism of underwater explosions was systematically clarified using the benchmarked FE model, and a parametric analysis was conducted to study the influence of steel grade, plate thickness, detonation distance, and the shape and position of charges on the dynamic behavior of steel plates subjected to underwater explosions. Based on the obtained results from both experiments and FE analyses, the coupling effect of the underwater explosion was systematically investigated, which provided a great basis for evaluating the local damage to steel structures and ships subjected to underwater explosions.

## 2. Experimental Program

### 2.1. Overview of Test

**Figure 1:**
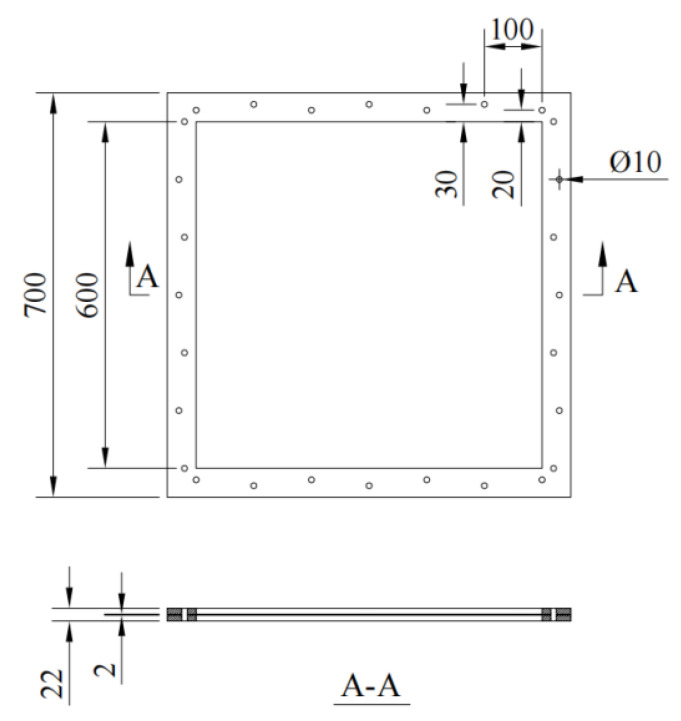
Schematic view of fixed steel sheet (unit: mm).

In this study, three underwater explosion tests were carried out to investigate the dynamic response of a fixed square steel sheet. The test system was designed according to the literature [3,4,9]. A trinitrotoluene (TNT) charge was employed, with charge weights (*W*) of 2.5 g, 5 g, and 10 g, respectively. The detonation distance (*R*) was 100 mm for all three tests. The 2.5 g TNT charge was a cylinder with a diameter of 12.5 mm and a height of 12.5 mm; the 5 g TNT charge was 15.8 mm in both diameter and height; the 10 g TNT charge was 19.8 mm in both diameter and height. The dimension of the square steel sheet (*B* × *B*) was 700 mm × 700 mm, and the thickness *(t*_s_) was 2 mm. A rigid steel frame was employed to constrain the steel sheet with bolted connections of 50 mm in width, as shown in Figure 1. Hence, the actual size of the tested steel sheet was 600 mm × 600 mm. The steel sheet was made of grade Q235, with a yield strength of 245 MPa and Young’s modulus of 206 GPa. Table 1 summarizes the parameters of all the tests.

### 2.2. Test Setup and Instruments

**Figure 2:**
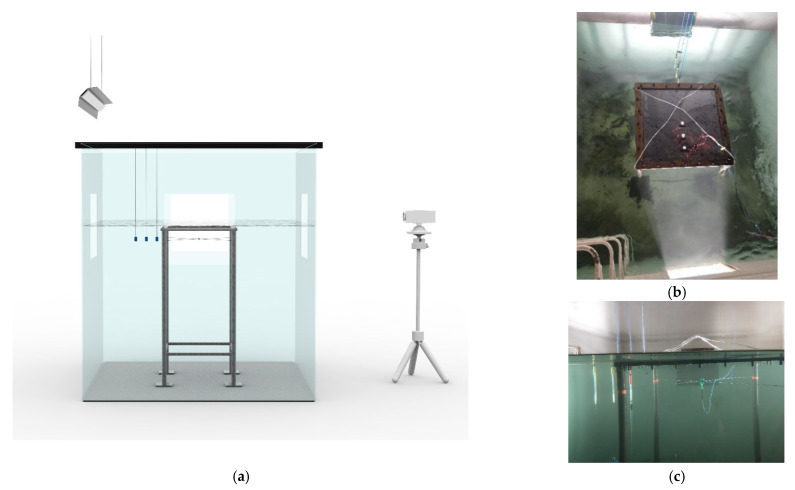
Overview of underwater explosion tests. (**a**) 3D view of water tank, specimen, and instruments. (**b**) Top view of specimen. (**c**) Front view of specimen.

The underwater explosion tests were conducted in a water tank, with dimensions of 2 m × 2 m × 2.2 m, as shown in Figure 2. The specimen was fixed on a steel frame with a height of 1.1 m, which was approximately located in the central area of the water tank, ensuring that the boundary of the water tank could be ignored. Four 30 kg weight blocks were applied on the four feet of the steel frame to ensure the fixed boundaries. The TNT charge was strapped to the steel frame at a detonation distance of 10 cm below the center of the tested steel sheet. No.8 electric detonators and an electric igniter were used. Three underwater pressure sensors PCB 138A-5a were used to record the dynamic pressure force of shock waves. At the same height as the explosive, these pressure sensors were arranged at a distance of 0.4, 0.5, and 0.6 m from the explosive (see Figure 2), which were connected through the cables to a signal conditioning and data acquiring system from National Instruments (NI). A high-speed camera was also adopted to monitor the explosion process through the observation port of the water tank.

## 3. Test Results and Discussion

### 3.1. Process of Underwater Explosion

**Figure 3:**
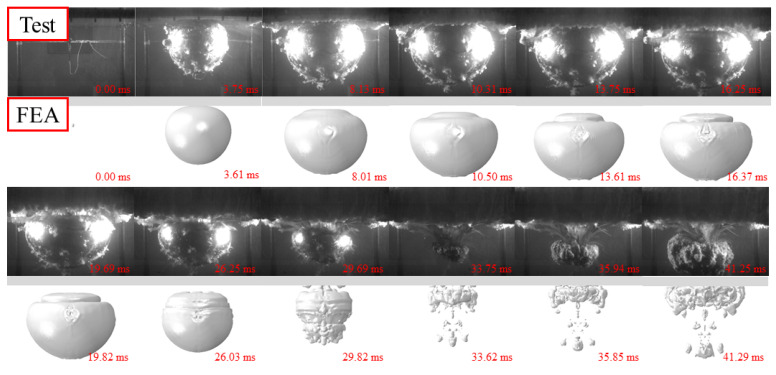
Whole process of underwater explosion for 2.5 g TNT.

**Figure 4:**
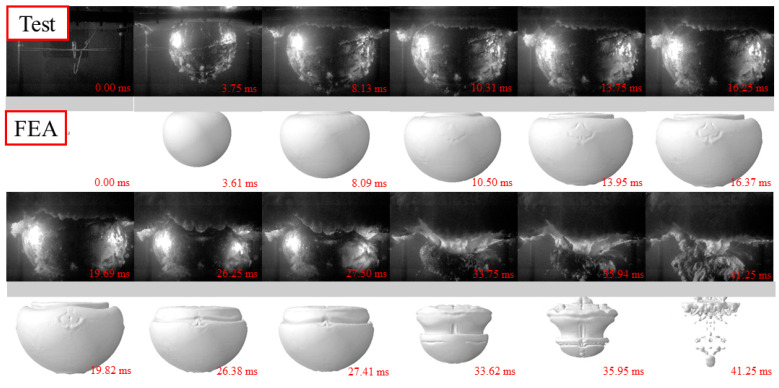
Whole process of underwater explosion for 5 g TNT.

**Figure 5:**
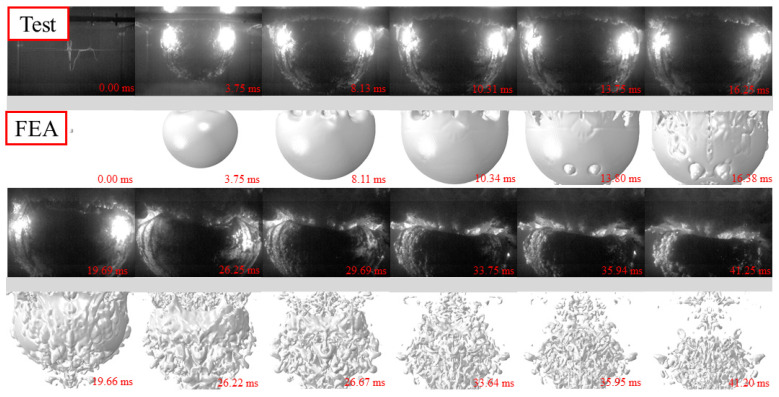
Whole process of underwater explosion for 10 g TNT.

Figure 3, Figure 4 and Figure 5 show the whole process of the underwater explosion, which was obtained from the high-speed camera during the experimental tests of 2.5 g, 5 g, and 10 g TNT charge. For various charge weights with the same detonation distances, the process of explosions was similar, showing the explosion starting, bubble expanding, and bubble collapsing. It should be noticed that the bubble size was greatly enlarged, and the time to reach the maximum bubble was also lagging, as the charge weight increased from 2.5 g to 10 g. This is because the released internal energy from a heavier charge is relatively larger than that from a lighter charge, resulting in larger pressure inside the bubble and larger time duration of explosion action.

The whole process of the underwater explosion was as follows. Taking the specimen with 2.5 g TNT as an example, the shock wave was rapidly formed and propagated outwards to the steel sheet after the explosion started at 0 ms. A bubble was also quickly generated and expanded after the explosion occurred, owing to the high pressure inside the bubble. The hogging deformation of the steel sheet was observed, owing to the shock wave, and at about 3.75 ms the hogging deformation typically reached the first peak. With the expansion of the bubble, the hogging deformation began to change to sagging deformation of the steel sheet, since the pressure inside the bubble (at the bottom of the steel sheet) was smaller than the ambient pressure of water (at the top of the steel sheet). When the bubble reached its maximum volume at about 16.25 ms, the steel sheet reached maximum sagging deformation, and the bubble began to shrink. At about 33.75 ms, the bubble basically collapsed and huge pressure was inside the bubble with a very small volume. Then, bubble pulsation was formed, leading to the obvious hogging deformation again of the steel sheet.

It should be noted that the time for reaching the complete collapse of the bubble for specimens with 10 g TNT was slightly earlier than that of specimens with 2.5 g and 5 g TNT. This was mainly because the deformation of the steel sheet was relatively large, and the steel plate protruded from the water’s surface and sucked in part of the air during the hogging process, which made the bubble collapse earlier.

### 3.2. Impulsive Pressure

**Figure 6:**
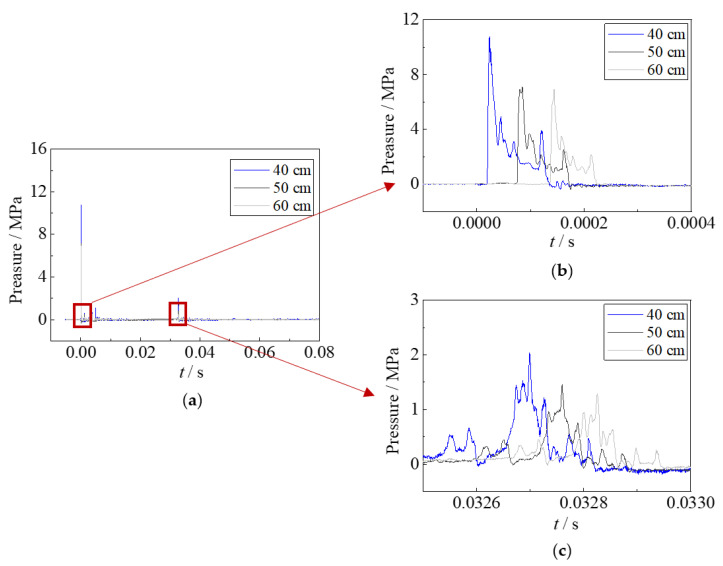
Impulsive pressure of underwater explosion for 2.5 g TNT. (**a**) Whole process. (**b**) Shock wave. (**c**) Bubble pulsation.

**Figure 7:**
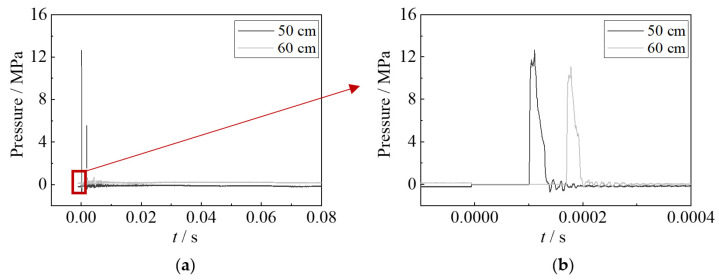
Impulsive pressure of underwater explosion for 5 g TNT. (**a**) Whole process. (**b**) Shock wave.

The impulsive pressure of underwater explosions was recorded by the pressure sensors. However, the impulsive pressure results of the specimen with 10 g TNT had not been collected, since the pressure sensors were damaged by the large shock wave. Figure 6 and Figure 7 show the impulsive pressure time–history curves of specimens with 2.5 g TNT and 5 g TNT. It showed that the impulsive pressures of shock waves at the distances of 0.4 m, 0.5 m, and 0.6 m away from the explosion point are 11.0 MPa, 7.2 MPa, and 6.8 MPa for 2.5 g TNT, respectively; the impulsive pressures at the distances of 0.5 m, and 0.6 m away from the explosion point are 12.5 MPa and 11 MPa for 5 g TNT, respectively. The impulsive pressure of 5 g TNT is about 1.7 times that of 2.5 g TNT, resulting in the larger deformation of specimens. The time duration of the impulsive pressure of the shock wave is relatively short, showing approximately 0.08 ms for 2.5 g TNT and 0.04 ms for 5 g TNT. It should be noted that the impulsive pressure on the steel sheet is much larger than those tested results, owing to the closer detonation distance of tests (i.e., 0.1 m).

The impulsive pressure of bubble pulsation was also recorded for the specimens with 2.5 g TNT. Although the sensors were far from the location of the explosion, resulting in a much smaller impulsive pressure than that at the specimen’s location, the bubble pulsation phenomenon was still clearly observed. The time duration of impulsive pressure of bubble pulsation is relatively large, showing 0.24 ms for 2.5 g TNT, which is approximately 3 times that of the shock waves. Hence, although the impulsive pressure of bubble pulsation is lower than that of the shock wave, the hogging deformation of the steel sheet is generated again.

### 3.3. Deformation

**Figure 8:**
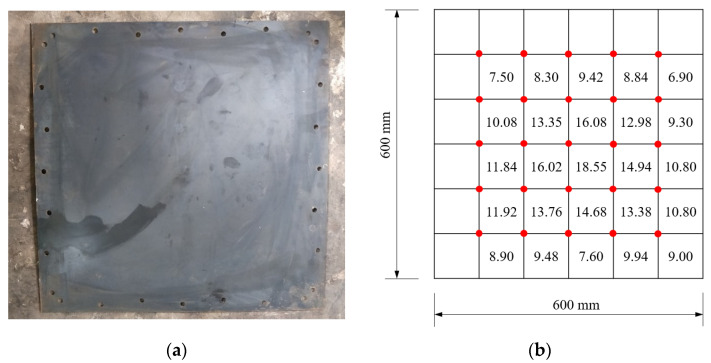
Deformation of the specimen with 2.5 g TNT. (**a**) Specimen after the explosion. (**b**) Deformation details.

**Figure 9:**
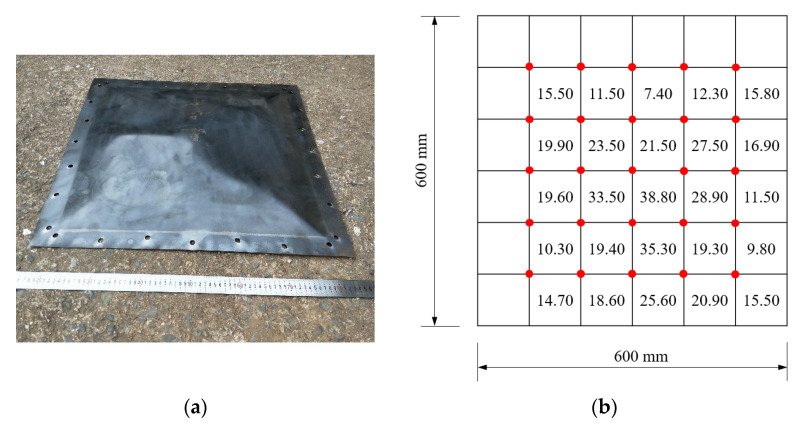
Deformation of the specimen with 5 g TNT. (**a**) Specimen after explosion. (**b**) Deformation details.

**Figure 10:**
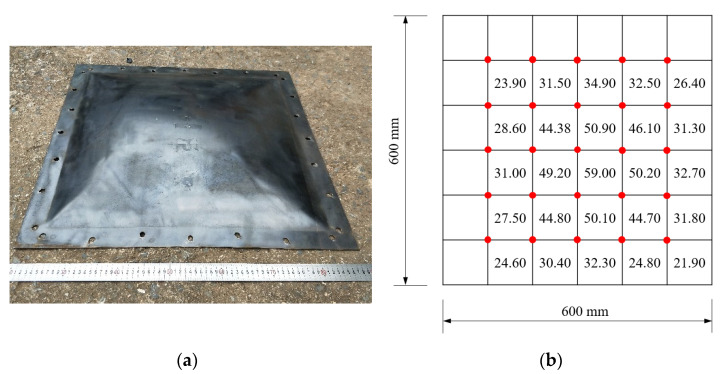
Deformation of the specimen with 10 g TNT. (**a**) Specimen after explosion. (**b**) Deformation details.

Figure 8, Figure 9 and Figure 10 show the residual deformation after testing for specimens with 2.5 g, 5 g, and 10 g TNT. All specimens present obvious hogging deformation after the explosion tests, showing a large deformation in the center of the steel sheet. As the charge weight increased, the maximum deformation was significantly increased, showing 18.55 mm, 38.8 mm, and 59.0 mm for specimens with 2.5 g, 5 g, and 10 g TNT, respectively.

## 4. Finite Element Analysis

### 4.1. Establishment of FE Model

#### 4.1.1. Details of FE Model

An FE model of underwater explosion tests was established using ABAQUS/Explicit. Generally, water, air, and charge were modeled using the Eulerian element (EC3D8R) by the Eulerian method, and the Eulerian volume fraction method to define the properties of all fluid materials in the element (i.e., water, air, and charge) was used in the Eulerian domain. The steel sheet was simulated using shell elements (S4R) by the Lagrange method. Hence, a coupled Eulerian–Lagrangian (CEL) method [28] combining the advantages of both the Euler and Lagrange methods was utilized in this study.

**Figure 11:**
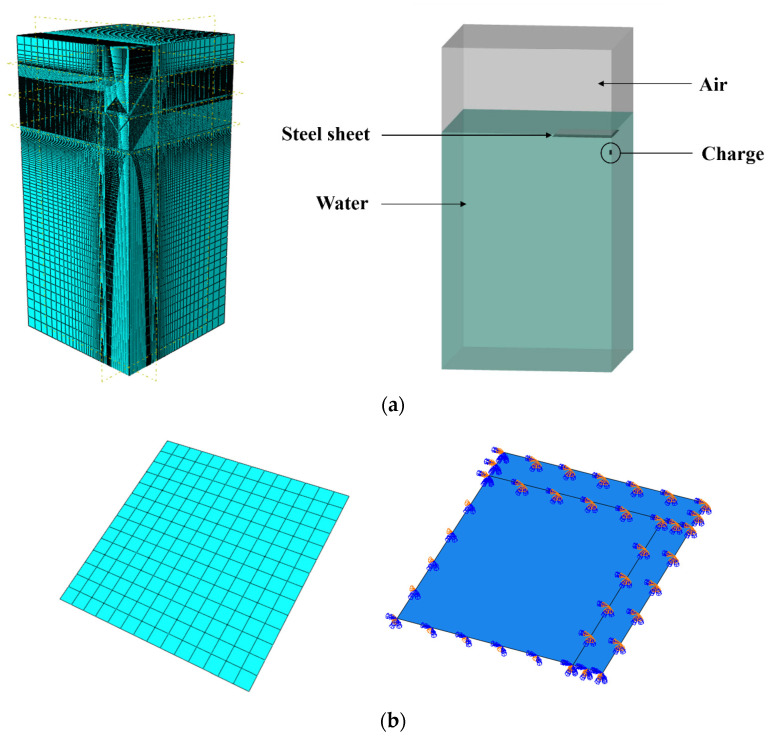
FE model of underwater explosion tests. (**a**) Eulerian elements (air, water, and charge). (**b**) Steel sheet.

**Figure 12:**
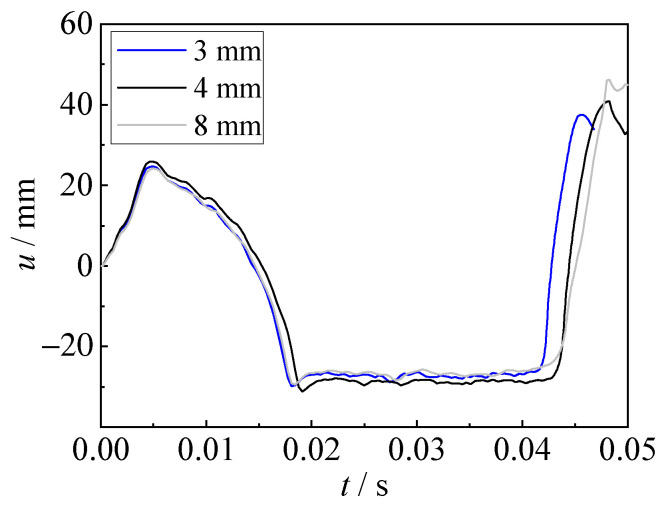
Mesh sensitivity analyses with various mesh sizes for the Eulerian domain.

To save the calculation resources, 1/4 of the model was established, as shown in Figure 11. The mesh size of Eulerian elements at the explosion area (i.e., the distance is 400 mm away from both the top, bottom, left, and right sides of the explosive) was 4 mm, and a gradient mesh was used outside the explosion area, with a maximum size of 150 mm. There are approximately 5 million elements for the Eulerian domain. The square steel sheet was modeled with a mesh size of 25 mm. It should be noted that the mesh sensitivity analyses with different mesh sizes (3 mm, 4 mm, and 8 mm) for the Eulerian domain were conducted. As shown in Figure 12, the predicted displacements are similar for models with various mesh sizes, and the maximum displacement of the model with an 8 mm mesh size is slightly larger than those of 3 and 4 mm mesh sizes. In addition, the costed time is 10, 24, and 48 h (using AMD EPYC 7643 with 48 cores) for mesh sizes of 8 mm, 4 mm, and 3 mm, respectively. Hence, the mesh size of 4 mm was adopted, considering the prediction accuracy and cost efficiency. Symmetrical boundary conditions (XSYMM, YSYMM) were set at the symmetrical surface, and fixed boundaries were set on the remaining surfaces. The fixed region of the steel sheet with 50 mm in width was also established in the FE model, which has an effect on the generation and collapse of the bubble.

#### 4.1.2. Equation of State

(1) Water

For water, the Mie–Grüneisen equation of state was adopted in ABAQUS, as shown in Equation (1), which assumed that the pressure of water (*p*) is a function of the density (*ρ*) and the internal energy of the unit mass.

(1) $$p-p_{H}=\Gamma \rho (E_{m}-E_{H})$$

where *p*_H_ is the Hugoniot pressure; *E*_H_ is the Hugoniot energy; *E*_m_ is the specific internal energy per unit mass; $\Gamma =\Gamma_{0}\rho_{0}/\rho$ is the Mie–Grüneisen coefficient.

The relationship between *E*_H_ and *p*_H_ can be expressed in Equation (2).

(2) $$E_{H}=\frac{p_{H}\eta }{2\rho_{0}}$$

where $\eta =1-\rho_{0}/\rho$, and *ρ*_0_ is the reference density. *p*_H_ can be given by:

(3) $$p_{H}=\frac{\rho_{0}c_{0}^{2}\eta }{{\left(1-s\eta \right)}^{2}}$$

Assuming the velocity of shock wave *U*_s_ and the velocity of particle *U*_p_ have a linear relationship, i.e., *U*_s_ = *c*_0_ + *sU*_p_, the Mie–Grüneisen equation can be presented in Equation (4).

(4) $$p=\Gamma_{0}\rho_{0}E_{m}+\frac{\rho_{0}c_{0}^{2}\eta }{{\left(1-s\eta \right)}^{2}}(1-\frac{\Gamma_{0}\eta }{2})$$

where *c*_0_ is the speed of sound; *Γ*_0_ is a material constant, and *s* is a constant in the *U*_s_–*U*_p_ equation. In this study, the density of water *ρ*_water_ is 1000 kg/m^3^, the speed of sound in water *c*_0_ is 1450 m/s, and *s* = 0 is adopted [28].

(2) Air

The ideal gas equation of state for air was adopted, as expressed in Equation (5).

(5) $$p+p_{A}=\rho R(\theta -\theta_{Z})$$

where *p*_A_ is the ambient pressure, *θ* is the current temperature, *θ*_Z_ is the temperature corresponding to absolute zero, and *R* is the gas constant (i.e., 287 J/kg/K) [28]. In this study, *p*_A_ is set to 101,300 Pa, and the density of air is 1.225 kg/m^3^ [28].

(3) Detonation

The well-accepted equation of state (i.e., JWL [29]) for detonation products was employed, as shown in Equation (6).

(6) $$p=A(1-\frac{\omega \rho }{R_{1}\rho_{c}})\exp(-R_{1}\frac{\rho_{c}}{\rho })+B(1-\frac{\omega \rho }{R_{2}\rho_{c}})\exp(-R_{2}\frac{\rho_{c}}{\rho })+\omega \rho E_{m}$$

where *A*, *B*, *R*_1_, *R*_2_, and ω are the material parameters, *ρ*_c_ is the density of charge, and *ρ* is the density of the detonation product. The material parameters in this study are defined as follows [28]: *ρ*_0_ = 1630 kg/m^3^, *A* = 3.7377 × 1011 Pa, *B* = 3.7471 × 109 Pa, *R*_1_ = 4.15, *R*_2_ = 0.9, *ω* = 0.35, *E*_m_ = 3.8 × 106 J/kg. In addition, the detonation velocity of the TNT charge is 6930 m/s [29].

#### 4.1.3. Dynamic Constitutive Model of Steel

Referring to previous studies [30], a continuous dynamic constitutive model of steel considering the influence of both strain rate and yield strength was employed in the FE model to depict the dynamic stress–strain curve across wide ranges of steel grades at various strain rates, as shown in the Equations (7)–(14). Note that the static stress–strain relationship model (i.e., Equation (8)) should be converted to the true plastic stress–strain curve.

(7) $$\sigma =\sigma_{s}(\varepsilon )\cdot {\mathrm{DIF}}_{\mathrm{avg}}(\dot{\varepsilon },f_{y})$$

(8) $$\sigma_{s}(\varepsilon )=\left\{ \begin{array}{l} E\varepsilon \\ f_{y}\\ f_{y}+(f_{u}-f_{y})\left\{0.4\varepsilon^{*}+\frac{2\varepsilon^{*}}{{\left[1+400{\left(\varepsilon^{*}\right)}^{5}\right]}^{0.2}}\right\} \end{array}\right.$$

(9) $$\varepsilon^{*}=\frac{\varepsilon -\varepsilon_{\mathrm{sh}}}{\varepsilon_{u}-\varepsilon_{\mathrm{sh}}}$$

(10) $$\varepsilon_{u}=0.6(1-\frac{f_{y}}{f_{u}}),\;\mathrm{but}\;\varepsilon_{u}\geq 0.06\;\mathrm{for}\;\mathrm{hot-rolled}\;\mathrm{steel}$$

(11) $$\varepsilon_{\mathrm{sh}}=0.1\frac{f_{y}}{f_{u}}-0.055,\;\mathrm{but}\;0.015\leq \varepsilon_{\mathrm{sh}}\leq 0.03$$

(12) $${\mathrm{DIF}}_{\mathrm{avg}}(\dot{\varepsilon },f_{y})=1+{\left(\frac{\dot{\varepsilon }}{D_{\mathrm{avg}}}\right)}^{\frac{1}{p_{\mathrm{avg}}}}$$

(13) $$D_{\mathrm{avg}}=1000{\left(\frac{f_{y}}{235}\right)}^{6}$$

(14) $$p_{\mathrm{avg}}=3{\left(\frac{f_{y}}{235}\right)}^{0.2}$$

where *σ* and *ε* are the stress and strain, respectively, *f*_y_, *f*_u_, and *E* are the yield strength, ultimate strength, and Young’s modulus of steel, respectively. $\varepsilon_{u}$ and $\varepsilon_{\mathrm{sh}}$ are the ultimate strain and strain-hardening strain of steel. $\dot{\varepsilon }$ is the strain rate.

### 4.2. FE Model Benchmarking

The whole process of the underwater explosion was also obtained by the FE analysis. Figure 3, Figure 4 and Figure 5 also compare the whole process of the underwater explosion from the high-speed camera during the experimental tests and the FE analysis for specimens with 2.5 g, 5 g, and 10 g TNT charges. It shows that the process of explosions is similar for tests and numerical results, and the periods of explosion starting, bubble expanding, and bubble collapsing have approximately coincided. Moreover, the bubble dimensions of tests and FE analysis are also similar.

**Figure 13:**
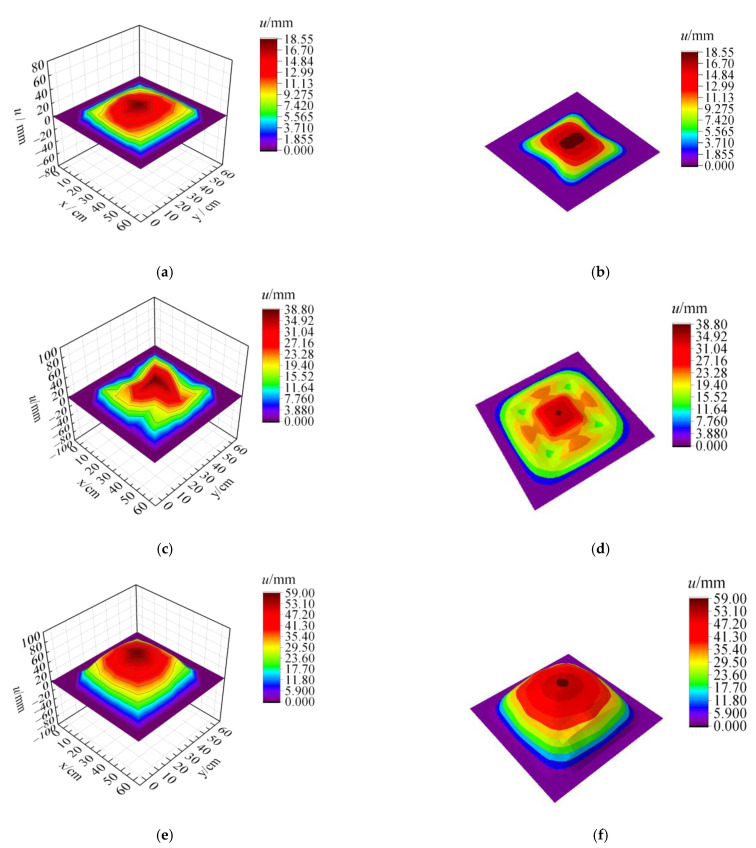
Comparison of deformation distribution between tests and FEA. (**a**) Measured results from tests (2.5 g TNT). (**b**) Predicted results from FEA (2.5 g TNT). (**c**) Measured results from tests (5 g TNT). (**d**) Predicted results from FEA (5 g TNT). (**e**) Measured results from tests (10 g TNT). (**f**) Predicted results from FEA (10 g TNT).

**Figure 14:**
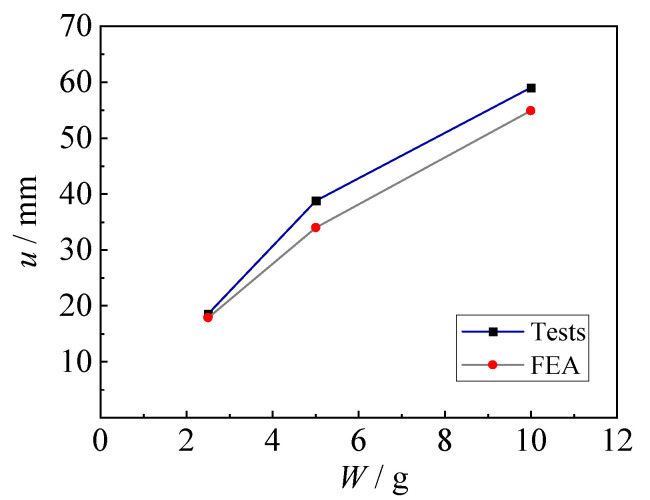
Comparison of maximum deformation between tests and FEA.

The failure mode of the fixed steel sheet after the underwater explosion obtained by the FE analysis was in good agreement with the test results. As shown in Figure 13, the specimens exhibit an obvious hogging deformation, where the maximum deformation appears in the central area. The deformation distributions between the test results and numerical results are almost consistent, with the variation of charge weights. Figure 14 shows the comparison of maximum deformation between tests and FEA, illustrating a great agreement. The predicted values from FEA for specimens with 2.5 g, 5 g, and 10 g TNT were 17.9 mm, 33.9 mm, and 54.9 mm, respectively, which only had less than 13% deviation from the test results. Hence, the established FE model in this study can reasonably predict the dynamic behavior of steel sheets subjected to underwater explosions.

### 4.3. Mechanism of Underwater Explosion

**Figure 15:**
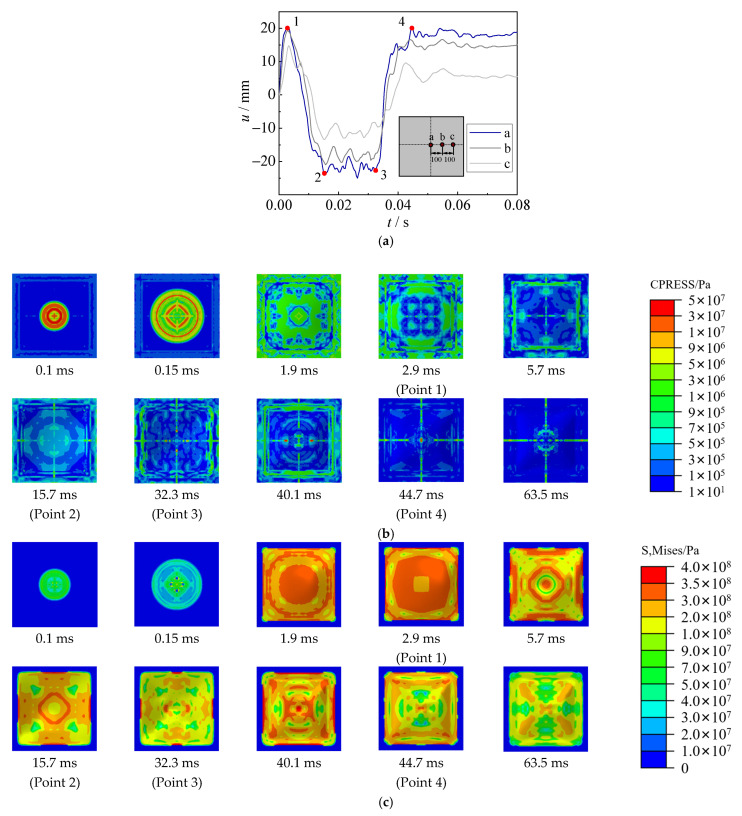
Displacement and stress development of specimens with 2.5 g TNT. (**a**) Displacement time–history curve. (**b**) Contact stress (CPRESS). (**c**) von Mises stress of steel sheet (S, Mises).

**Figure 16:**
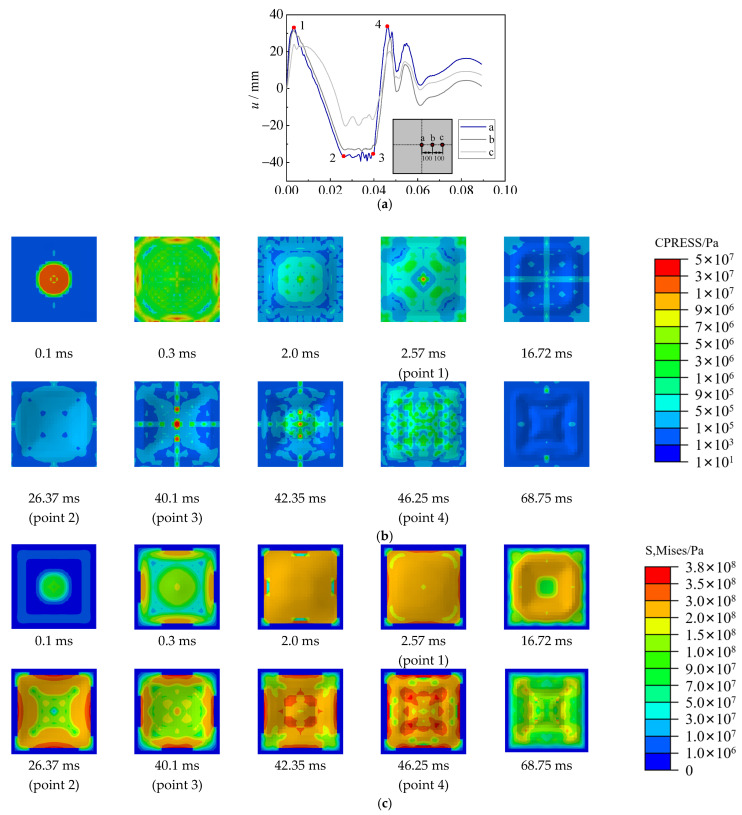
Displacement and stress development of specimens with 5 g TNT. (**a**) Displacement time–history curve. (**b**) Contact stress (CPRESS). (**c**) von Mises stress of steel sheet (S, Mises).

**Figure 17:**
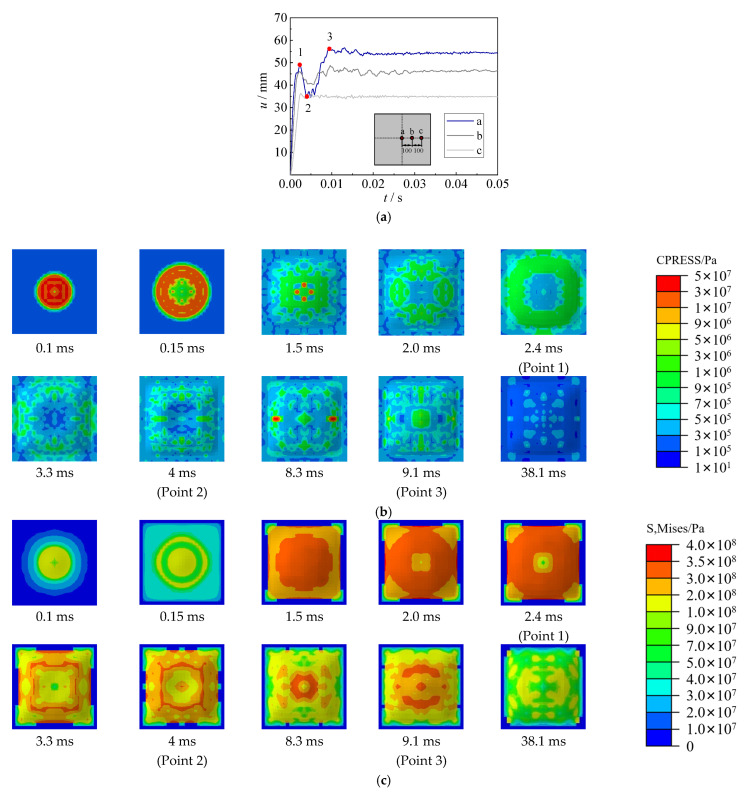
Displacement and stress development of specimens with 10 g TNT. (**a**) Displacement time–history curve. (**b**) Contact stress (CPRESS). (**c**) von Mises stress of steel sheet (S, Mises).

Based on the benchmarked FE model, the displacement time–history curve, contact stress between the steel sheet and water, and von Mises stress of the steel sheet have been analyzed in the section. Figure 15, Figure 16 and Figure 17 show the displacement and stress development of specimens with 2.5 g TNT, 5 g TNT and 10 g TNT. In these figures, the a, b, and c points are the central point of the steel sheet, 100 mm away from the central point, and 200 mm away from the central point, respectively.

For the specimen with 2.5 g TNT, the deformation development process of hogging, sagging, and hogging again was observed in Figure 15a. Large contact stress (about 50 MPa) between the steel sheet and water was observed in Figure 15b, which is mainly due to shock waves at the early stage of underwater explosions (0.1 ms). The deformation was quickly increased to 20 mm at 2.9 ms, and the steel sheet yielded in most areas (see Figure 15c). Subsequently, owing to the pressure inside the bubble (at the bottom of the steel sheet) being smaller than the ambient pressure of water (at the top of the steel sheet), a sagging deformation began to be generated, and the contact stress was reduced to a relatively low level. At about 32.3 ms (Point 3), bubble pulsation was formed when the bubble collapsed. At this time, the contact stress in some areas was also increased to approximately 50 MPa, leading to the hogging deformation again. It should be noted that owing to the large time duration of bubble pulsation, the deformation caused by bubble pulsation may be larger than that by the shock waves. Due to the combined effect of the hogging deformation and the sagging deformation, the distribution of von Mises stress was not uniform when the maximum deformation was reached (Point 4). The maximum stresses were concentrated on the center and supports of the steel sheet. For the specimen with 5 g TNT, the process of deformation development, contact stress, and von Mises stress are similar to those of 2.5 g TNT, as shown in Figure 16.

For the specimen with 10 g TNT, the deformation development process of hogging, sagging, and hogging again was also observed in Figure 17a. However, the deformation of the steel sheet was relatively large, some air had been sucked into the bubble during the hogging process, which made the bubble collapse earlier and result in a relatively lower sagging deformation. Hence, only the central area of the steel sheet showed sagging deformation (i.e., points a and b in Figure 17a); point c showed no sagging deformation. After point 3 in Figure 17, the deformation was basically kept unchanged, and the contact stress was reduced to a low level.

### 4.4. Parametric Analysis

To further investigate the dynamic behavior of fixed steel sheets subjected to underwater explosions, a parametric analysis was conducted systematically herein, with the variations of charge weight *W* (5 g and 10 g), steel grade (Q235, Q355, Q460, Q550, and Q690), thickness of steel sheet *t*_s_ (1 mm, 2 mm, 4 mm, 6 mm, 8 mm, and 10 mm), detonation distance *R* (60 mm, 100 mm, 200 mm, 300 mm, 400 mm, and 500 mm), and shape and position of charges (i.e., the length-to-diameter ratios are 1:1, 5:1, and 10:1; the cylinder TNT charges are arranged vertically or transversely, marked as V and T, respectively). Other parameters remained unchanged, i.e., the dimension of the steel sheet *B* × *B* is 600 × 600 mm, and the boundary is all fixed. The employed parameters were typically based on the tests, and considered the advances in materials and possible application in actual engineering structures and ships. Table 2 summarizes the detailed information.

#### 4.4.1. Effect of Steel Grade

**Figure 18:**
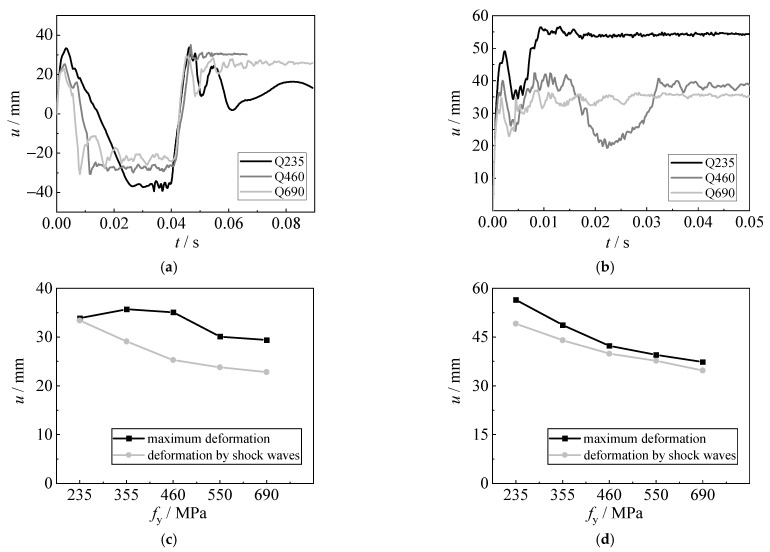
Effect of steel grade. (**a**) Displacement time histories (5 g TNT). (**b**) Displacement time histories (10 g TNT). (**c**) Deformation (5 g TNT). (**d**) Deformation (10 g TNT).

Figure 18 shows the influence of steel grade (Q235, Q355, Q460, Q550, and Q690) on the displacement time histories, the deformation caused by shock waves, and the maximum deformation of the steel sheet. As the yield strength of the steel sheet increases, the deformation caused by shock waves is gradually reduced, as the flexural resistance is improved when using high-strength steel. Under the explosions with 5 g TNT, the sagging deformation is obvious after the first shock wave, and then the bubble pulsation significantly increases the hogging deformation. Although the yield strength of the steel sheet increases, the maximum deformation caused by the bubble pulsation is slightly reduced. For the explosions with 10 g TNT, the hogging deformation caused by shock waves is relatively larger, and the sagging deformation is not obvious. The maximum deformation caused by bubble pulsation is typically lower than those caused by shock waves, especially for high-strength steels. Using higher-strength steel can reduce the deformation and improve the capacity of the steel sheet subjected to underwater explosions.

#### 4.4.2. Effect of Plate Thickness

**Figure 19:**
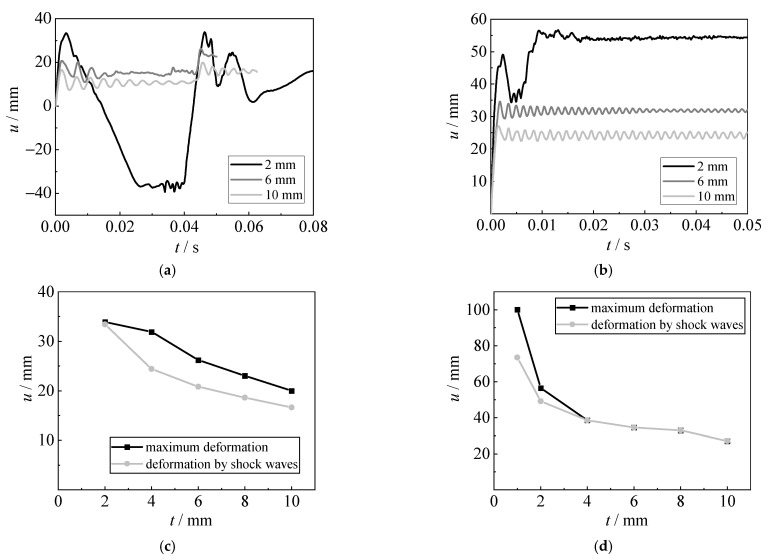
Effect of plate thickness. (**a**) Displacement time histories (5 g TNT). (**b**) Displacement time histories (10 g TNT). (**c**) Deformation (5 g TNT). (**d**) Deformation (10 g TNT).

Figure 19 shows the influence of plate thickness (1 mm, 2 mm, 4 mm, 6 mm, 8 mm, and 10 mm) on the displacement time histories, the deformation caused by shock waves, and the maximum deformation of the steel sheet. As the plate thickness increases, the sagging deformation becomes less obvious. For the explosion with 5 g TNT, the hogging deformation caused by bubble pulsation is slightly larger than those caused by shock waves. When the plate thickness is larger than 4 mm, the hogging deformation caused by bubble pulsation is basically lower than those caused by shock waves for 10 g TNT. The maximum deformation is reduced with the increase in plate thickness. Using thicker steel sheets can reduce the deformation and improve the capacity of the steel sheet subjected to underwater explosions, especially for the deformation caused by bubble pulsation.

#### 4.4.3. Effect of Detonation Distance

**Figure 20:**
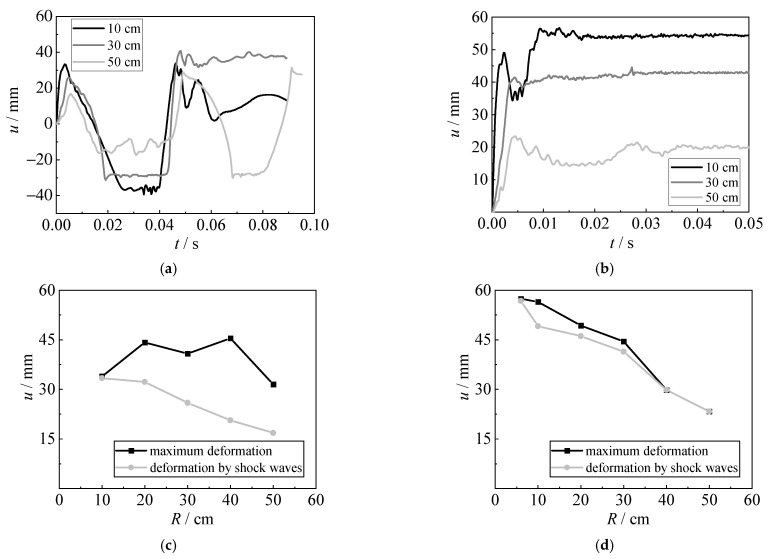
Effect of detonation distance. (**a**) Displacement time histories (5 g TNT). (**b**) Displacement time histories (10 g TNT). (**c**) Deformation (5 g TNT). (**d**) Deformation (10 g TNT).

Figure 20 shows the influence of detonation distance (6 cm, 10 cm, 20 cm, 30 cm, 40 cm, and 50 cm) on the displacement time histories, the deformation caused by shock waves, and the maximum deformation of the steel sheet. As the detonation distance of the underwater explosion increases, the deformation caused by shock waves is gradually reduced, since the shock waves are significantly decreased. For the explosion with 5 g TNT, the hogging deformation caused by bubble pulsation is larger than those caused by shock waves, and the sagging deformation also becomes smaller. When the detonation distance of the underwater explosion increases to the bubble radius, the action of bubble pulsation becomes larger, leading to the larger deformation of the steel sheet; the deformation is also reduced when the detonation distance is further increased. Owing to the air sucked inside the bubble, the bubble pulsation is reduced for explosions with 10 g TNT, resulting in similar deformations caused by shock waves and bubble pulsation.

#### 4.4.4. Effect of Shape and Position of the Charge

**Figure 21:**
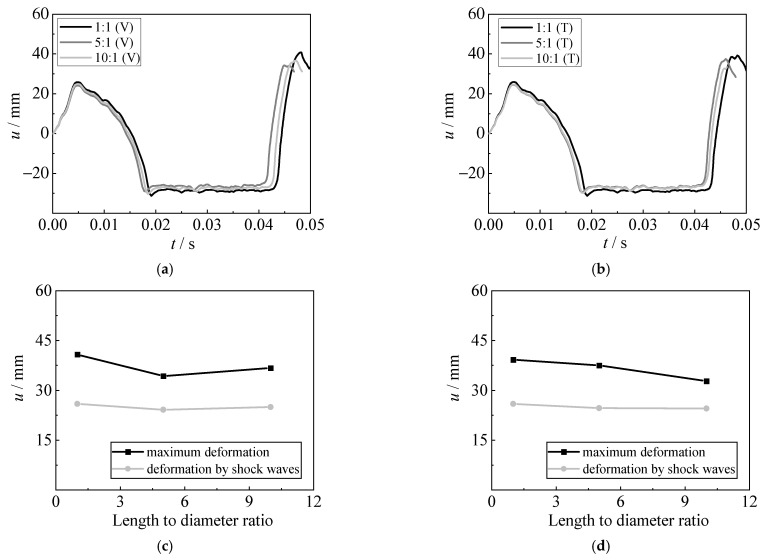
Effect of shape and position of the charge. (**a**) Displacement time histories (Vertically). (**b**) Displacement time histories (Transversely). (**c**) Deformation (Vertically). (**d**) Deformation (Transversely).

**Figure 22:**
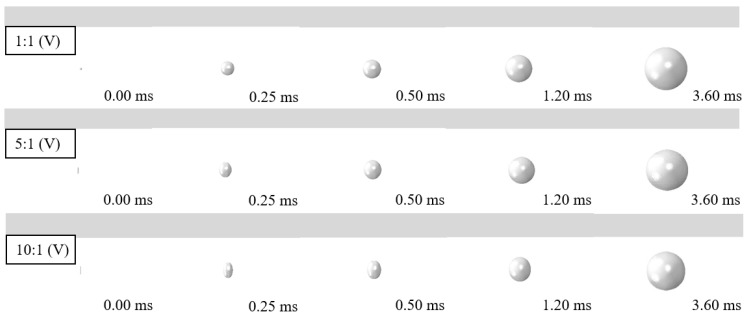
Comparison of the bubble development for various shapes of the charge.

Figure 21 shows the influence of shape and position of charges (the length-to-diameter ratios are 1:1, 5:1, and 10:1; the cylinder TNT charges are arranged vertically or transversely) on the displacement time histories, the deformation caused by shock waves, and the maximum deformation of the steel sheet. It shows that the shape of the charge has a slight influence on the displacement of a fixed steel sheet subjected to underwater explosions. As the length-to-diameter ratio of the charge increases, the deformation caused by both shock waves and bubble pulsation is slightly reduced. In addition, the deformations were almost similar when the charge was arranged vertically or transversely. Figure 22 compares the bubble development for various shapes of the charge. It shows that at the early stage of the underwater explosion (e.g., 0.25 ms), the bubble presents an ellipsoid when the length-to-diameter ratio increases to 5:1 and 10:1. However, the bubble shows a similar sphere at 3.6 ms, although the length-to-diameter ratio varies. This is the main reason for the limited influence on the dynamic behavior of steel sheets subjected to various shapes and positions of the charge. Nevertheless, the charge with a 1:1 length-to-diameter ratio which is arranged vertically still causes a relatively large blast effect, resulting in slightly larger deformation of the steel sheets.

## 5. Conclusions

Three underwater explosion tests with three different TNT charge weights (i.e., 2.5 g, 5 g, and 10 g) were conducted to investigate the dynamic response of a fixed square steel sheet. A FE model was established and benchmarked based on the test results. The deformation development and stress mechanism of underwater explosions have been analyzed experimentally and numerically. Several conclusions can be drawn as follows:

1. The steel sheet shows a process of hogging deformation, sagging deformation, and hogging deformation again, due to the actions of shock waves, bubble expansion, bubble collapse, and bubble pulsation. The air may be sucked into the bubble during the hogging process, making the bubble collapse earlier and resulting in a relatively lower sagging deformation for the explosions with a large charge weight of TNT.
2. The time duration of the impulsive pressure of bubble pulsation is relatively large, which is approximately three times that of shock waves. Although the impulsive pressure of bubble pulsation is lower than that of shock waves, the deformation caused by bubble pulsation is larger than that by the shock waves, owing to the large time duration of bubble pulsation.
3. The damage to the steel plate gradually decreases, with the increase in steel strength, plate thickness, and detonation distance. Using higher-strength steel and thicker steel sheet can reduce the deformation and improve the blast capacity of the steel sheet, especially for the deformation caused by bubble pulsation. The action of bubble pulsation becomes larger when the detonation distance increases to bubble radius (about 40 cm), resulting in the largest deformation. A limited influence on the dynamic behavior of steel sheets subjected to various shapes and positions of the charge was observed.

## Figures and Tables

**Table 1:** Summary of test parameters.

No.	Specimen Dimension (*B* × *B* × *t*_s_ mm)	Steel Grade	Charge Type	*R* (mm)	*W* (g)
1	600 × 600 × 2	Q235	TNT	100	2.5
2					5
3					10

**Table 2:** Summary of test parameters.

No.	Parameters	Dimensions (*B* × *B* mm)	Length-to-Diameter Ratio of the Charge	*W* (g)	Steel Grade	Thickness *t*_s_ (mm)	*R* (mm)
1	Steel grade	600 × 600	1:1 (V)	10	Q235	2	100
2					Q355		
3					Q460		
4					Q550		
5					Q690		
6			1:1 (V)	5	Q235	2	100
7					Q355		
8					Q460		
9					Q550		
10					Q690		
11	Thickness	600 × 600	1:1 (V)		Q235	1	100
12						4	
13				10		6	
14						8	
15						10	
16			1:1 (V)	5	Q235	4	100
17						6	
18						8	
19						10	
20	Detonation distance	600 × 600	1:1 (V)	10	Q235	2	60
21							200
22							300
23							400
24							500
25			1:1 (V)	5	Q235	2	200
26							300
27							400
28							500
29	Length-to-diameter ratio	600 × 600	1:1 (T)	5	Q235	2	300
30			5:1 (V)				
31			5:1 (T)				
32			10:1 (V)				
33			10:1 (T)				

## Data Availability

Data are available from the corresponding author and can be shared with anyone upon reasonable request.
